# Beat Patterns Determine Inter-Hand Differences in Synchronization Error in a Bimanual Coordination Tapping Task

**DOI:** 10.1177/20416695211053882

**Published:** 2021-10-29

**Authors:** Yuka Saito, Tomoki Maezawa, Jun I. Kawahara

**Affiliations:** Department of Psychology, 12810 Hokkaido University, Sapporo, Japan

**Keywords:** negative mean asynchrony, synchrony, bimanual coordination, tapping, handedness

## Abstract

A previous study reported the unique finding that people tapping a beat pattern with the right hand produce larger negative synchronization error than when tapping with the left hand or other effectors, in contrast to previous studies that have shown that the hands tap patterns simultaneously without any synchronization errors. We examined whether the inter-hand difference in synchronization error occurred due to handedness or to a specificity of the beat pattern employed in that study. Two experiments manipulated the hand–beat assignments. A comparison between the identical beat to the pacing signal and a beat with a longer interval excluded the handedness hypothesis and demonstrated that beat patterns with relatively shorter intervals were tapped earlier (Experiment 1). These synchronization errors were not local but occurred consistently throughout the beat patterns. Experiment 2 excluded alternative explanations. These results indicate that the apparent inconsistency in previous studies was due to the specificity of the beat patterns, suggesting that a beat pattern with a relatively shorter interval between hands is tapped earlier than beats with longer intervals. Our finding that the bimanual tapping of different beat patterns produced different synchronization errors suggests that the notion of a central timing system may need to be revised.

## Introduction

We often synchronize our body movements to external periodic stimuli (e.g., our clapping is synchronized to music). This phenomenon, referred to as sensorimotor synchronization (SMS; Repp, 2005; Repp & Su, 2013), involves two independent motor systems, one for each hand, and a central timing system that governs these two systems (Hestermann et al., 2018; Pabst & Balasubramaniam, 2018; Vorberg & Hambuch, 1984). SMS has been demonstrated using synchronization tapping tasks in which participants synchronize external stimuli with movements of unilateral (Aschersleben & Prinz, 1995; Nalηacı et al., 2001) and bilateral motor effectors (Aschersleben & Prinz, 1995; Fujii et al., 2011; Vorberg & Hambuch, 1984; Yamanishi et al., 1980). In this task, synchronization error (SE), defined as the time difference between the onset of an external stimulus and the time at which a finger hit the table, is a critical measure of synchronous tapping movements.

The SE tends to be negative, so participants tap ahead of the external periodic stimulus (pacing signal) by a few tens of milliseconds. This tendency is known as negative mean asynchrony (NMA) (Repp, 2005; Repp & Su, 2013). However, in one previous study, no differences in SE were found between bilateral hands in a synchronization tapping task that required participants to synchronize the tapping of fingers on both hands to a pacing signal (Aschersleben & Prinz, 1995).

**Figure 1.**
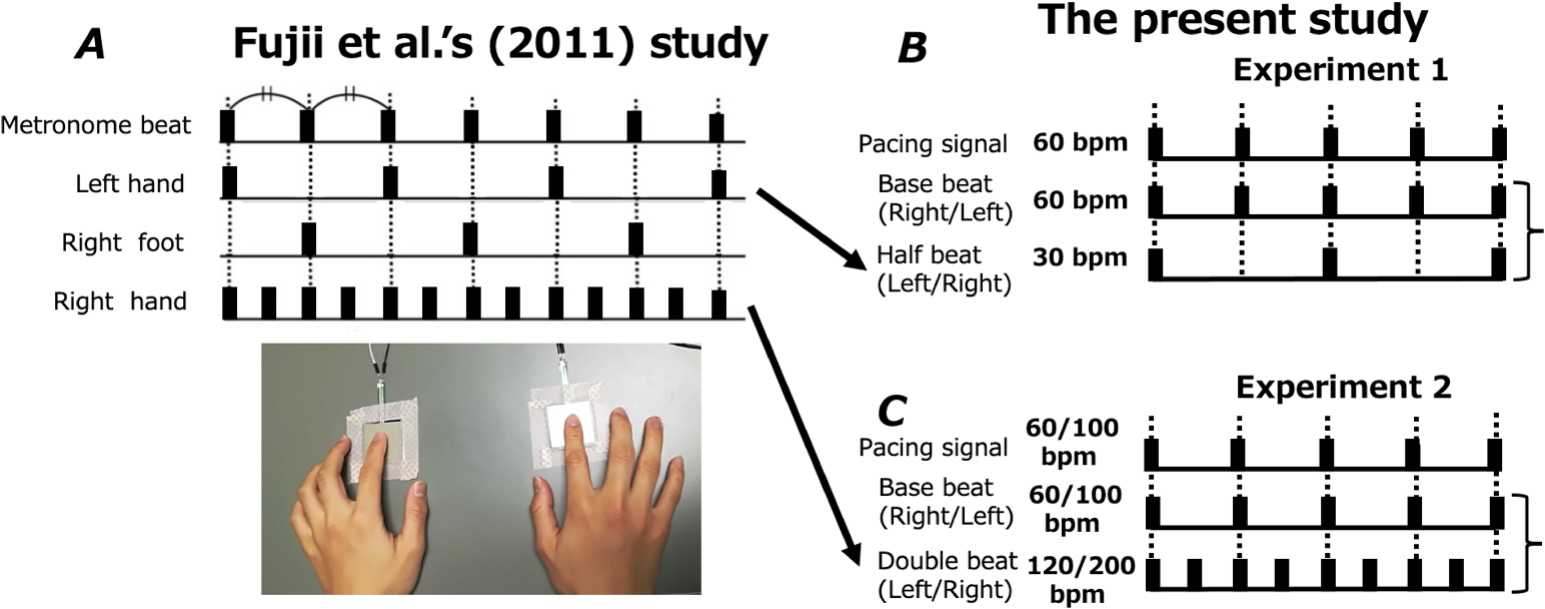
Beat patterns in (A) the Fujii et al. (2011) study and (B and C) in the present study (double-beat task and half-beat task). The first row of each panel in B and C shows the beat pattern of the auditory pacing signal, and the second row shows the base-beat pattern tapped at the same interval as the auditory pacing signal (Experiment 1). In the Fujii et al., study, the base beat was the combined beat pattern of the left hand and right foot. The third row of panel B is the half beat (one tap per two auditory signals) and the third row in panel C is the double beat (two taps per one auditory pacing signal (Experiment 2). Participants tapped the base beat and half beat to the pacing signal presented at 60 bpm using both hands or a single hand in Experiment 1. In Experiment 2, participants tapped the base beat and double beat using both hands in response to the pacing signal presented at 60 or 100 bpm.

By contrast, another study found that tapping two hands synchronously produced different mean SEs among three effectors. Specifically, Fujii et al. (2011) found a difference in SEs between the right and left hands when professional drummers tapped with their right hand, left hand, and right foot to synchronize to designated beats. In their study, the right hand tapped twice per auditory pacing signal, and the left hand and the right foot tapped once per two auditory pacing signals, alternating throughout the experimental session, as shown in Figure 1A. The results showed that the SEs of the left hand and right foot taps were equivalent and that those errors were larger than the errors of the right hand.

There are two possible interpretations of this difference in negative mean asynchrony between hands. The first explanation is based on handedness (the handedness hypothesis). Because all participants in the Fujii et al., study (2011) were right-handed, it is reasonable to assume that the dominant hand was attentionally prioritized over the non-dominant hand; thus, participants perceived the interval as shorter for the dominant hand, as demonstrated by Zakay (1993). This biased prioritization would cause greater SEs in the non-dominant hand relative to the dominant hand in Fujii et al. (2011). The second interpretation is based on characteristics of the beat pattern (the beat-pattern hypothesis). Specifically, Fujii et al. (2011) involved three effectors and used mixed beat patterns including one double (right hand) and two half inter-onset intervals (left hand and right foot) of the reference metronome beat. Because periodic hand movement synchronizing with a metronome reference can be disturbed by adding more frequent periodic responses (Walter et al., 1998), tapping under the mixed beat pattern used in Fujii et al. (2011) could have led to an increase in SEs. This disturbance may have contributed to the difference in the negative mean asynchrony between the two hands.

To determine whether either of these hypotheses could explain the difference in the SEs between the left and right hands in Fujii et al. (2011), we designed a novel set of experiments. In our experiments, we measured tapping responses while switching hands across experimental blocks to examine the contribution of dominant handedness. We reasoned that if handedness determined the difference in SEs between the left and right hands, the beat patterns tapped by the non-dominant hand should produce larger negative SEs than those tapped by the dominant hand regardless of the beat patterns to be tapped (i.e., the beat patterns tapped by the non-dominant hand would produce greater SEs than those tapped by the dominant hand even when those patterns were switched).

In Experiment 1, participants were asked to synchronize tapping to the base beat and half beat, with the auditory pacing signals presented at 60 bpm, using both hands (bimanual tapping condition) or a single hand (unimanual tapping control condition). In Experiment 2, participants were asked to synchronize tapping to the base beat and double beat, with the auditory pacing signals presented at 60 or 100 bpm, using both hands. The base beat was the same beat interval as the auditory pacing signal. The half beat pattern was tapped at half the tempo of the pacing signal, and the double beat pattern was tapped at twice the tempo of the pacing signal. When the tempo of the auditory pacing signal was 60 bpm, the tapping tempos were 30, 60, and 120 bpm under the half-beat, base-beat, and double-beat conditions, respectively. When the tempo of the auditory pacing signal was 100 bpm, the tapping tempos were 50, 100, 200 bpm under the half-beat, base-beat, and double-beat conditions, respectively. The half beat was halved from the beat of the longer inter-onset interval, similar to the left-hand beat in the Fujii et al. (2011) study, and the half beat was synchronized with the base beat (Figure 1B). The double beat was synchronized with the base beat, as in the right-hand beat of Fujii et al.*,* in Experiment 2 (Figure 1C).

## Experiment 1

As described above, the first experiment was designed to replicate Fujii et al.’s (2011) findings using a simplified beat pattern that excluded foot-tapping responses and allowed us to address two hypothetical explanations for the earlier results, namely, the handedness hypothesis and the beat-pattern hypothesis. We recruited novice participants to examine whether Fujii et al.'s findings would generalize to a broader range of participants (non-musicians), as all participants in the previous research were drummers (musicians). A unimanual tapping task was included as a control condition to assess the effect of bimanual tapping on SE. If tapping with both hands increased the SE, the SE for the bimanual tapping task should be larger than that of the unimanual tapping task. We used a 60 bpm tempo in this experiment, although Fujii et al. (2011) used three (60, 120, and 200 bpm). We chose this tempo because preliminary testing revealed that novice participants were unable to perform the task under any tempo over 120 bpm, whereas performance was optimal under the 60 bpm condition.

### Method

#### Participants

Nineteen Hokkaido University undergraduate and graduate students (mean age = 20.45 years, SD = 2.0, male:female = 14:5) participated. The sample size was determined by an estimate of desired statistical power (with f = 0.25 and β = 0.8, the optimal sample size was 16) using G*Power software v3.0 (Faul et al., 2007). This number was comparable to the number who participated in Fujii et al. (2011) (N = 15). Three participants were excluded from the analyses: two were unable to complete the task, and one mistakenly tapped the wrong beat pattern. All participants had never received musical training for more than 3 years, and all were right handed. All experiments reported in the present study were approved by the Human Research Ethics Committee of Hokkaido University, Japan, and we obtained written informed consent from participants prior to the experiment.

#### Materials and Stimuli

The auditory pacing signals were generated by a Raspberry Pi 4 (Model B, Raspberry Pi foundation, UK) via headphones (MDR-XB550, SONY, Japan) with a comfortable loudness level. The auditory pacing signals were presented at a rate of 60 bpm (inter-onset interval: 1,000 ms) or 100 bpm (inter-onset interval: 600 ms). Two force-sensing resistors (FSR-406; Interlink Electronics, Carmarillo, CA, USA) were connected to a single board computer (Raspberry Pi 4) to record the timing of participants’ tapping responses. The temporal resolution to acquire tap timing was 1,000 Hz. The difference between the periodicity of the presented auditory pacing signal and the ideal periodicity was basically under 10 μs maximum, and the average difference was 60 μs. Two LEDs connected to the Raspberry Pi 4 that lit up in time to the participants’ tapping responses were used to provide a continuous light-on signal as visual feedback. We did not use the drum sound employed by Fujii et al. (2011) to avoid interference of auditory feedback with the auditory pacing signal. The LEDs were separated by 1.5 cm and located 30 cm away from the seated participants.

#### Tasks and Conditions

**Figure 2.**
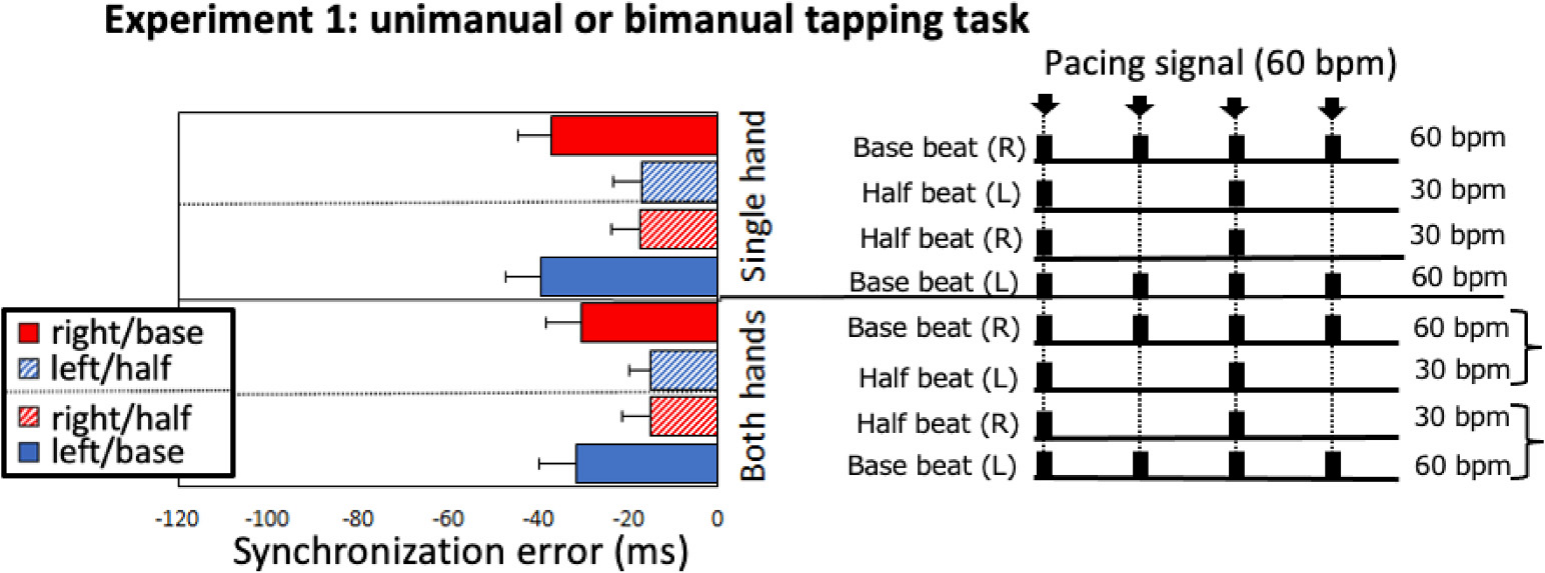
Synchronization errors (SE, ms) in Experiment 1. Left: SEs as a function of hand (both or single) and beat (base or half) for each effector (left or right). The error bars represent the standard error of the average SE. Right: Beat patterns corresponding to the bars in the left panel. Arrows indicate the onset of the pacing signal. The thick bars indicate the moment participants tapped the base beat at 60 bpm (and the double beat at 120 bpm) in response to the 60 bpm pacing signal.

Participants synchronized their finger-tapping responses with the auditory pacing signal under two hand–beat patterns (curly brackets in Figure 2) or four single hand–beat patterns († in Figure 2). Participants performed the bimanual tapping and unimanual tapping tasks in a random order. During the bimanual tapping task, the first block consisted of the base beat with the right hand and the half beat with the left hand. The second block consisted of the opposite hand–beat assignment (i.e., the base beat with the left hand and the half beat with the right hand). Half of the participants performed the two hand–beat patterns in this order, and the remaining half performed with the order reversed. During the unimanual tapping task, the four unimanual patterns were presented in random order for each participant. Regardless of the hand–beat pattern, participants tapped once per pacing signal for the base beat and once after every two pacing signals for the half beat.

#### Procedure

Participants sat in a chair in front of a desk in a soundproofed room and tapped on the force-sensing resisters with both index fingers; participants stared at the two LEDs throughout the task. Each block included four beats for beat extraction and 24 beats for beat production. During the beat extraction, participants counted four beats in their mind without tapping and then initiated tapping on the arrival of the fifth beat. The sequence of these 28 critical beats were considered a trial and were repeated eight times for each block. Except for their fingers, participants were instructed keep their bodies as immobile as possible to avoid producing the beat with other motor effectors during the task and to raise their fingers as soon as possible after tapping the force-sensing resistors. The moment of tap-down was measured by two force-sensing resistors. A tap was defined as the moment the pressure voltage surpassed a threshold, i.e., the lowest voltage at which no noise responses were detected during the resting state. A negative tap value indicated that the participant tapped before the auditory pacing signal was emitted, whereas a positive value indicated a tap after the auditory pacing signal was emitted.

### Results

All participants completed four unimanual and two bimanual tapping tasks. In the unimanual tapping task, the participants performed the base-beat condition using the right hand (SE: −36.87 ± 29.96 ms, [mean ± standard deviation]) or left hand (SE: −39.26 ± 31.33 ms) alone. Similarly, the half-beat condition was performed using the right hand (SE: −16.87 ± 30.96 ms) or left hand (SE: −17.23 ± 25.25 ms) alone. In the bimanual tapping task, participants performed two beat combinations: the base beat using the right hand (SE: −30.44 ± 30.96 ms) and half beat using the left hand (SE:−14.95 ± 18.81 ms), or the base beat using the left hand (SE: −31.38 ± 32.26 ms) and half beat using the right hand (SE: −15.13 ± 24.04 ms).

Regarding the mean SE between the auditory pacing signal and tapping response, the left panel of Figure 2 shows the mean SEs. We performed a three-way repeated measures analysis of variance (ANOVA) with SE as the dependent factor and hand (right vs. left), beat pattern (half beat vs. base beat), and tapping (unimanual vs. bimanual) as independent variables to investigate their effects on mean SE. The analysis revealed a significant main effect of beat pattern (*F* (1,15) = 33.20, *p* < 0.001, η_p_^2^ = 0.689) but no main effect of hand or tapping (hand: *F* (1,15) = 0.13, *p* = 0.720, η_p_^2^ = 0.009; tapping: *F* (1,15) = 2.72, *p* = 0.120, η_p_^2^ = 0.153).

**Figure 3.**
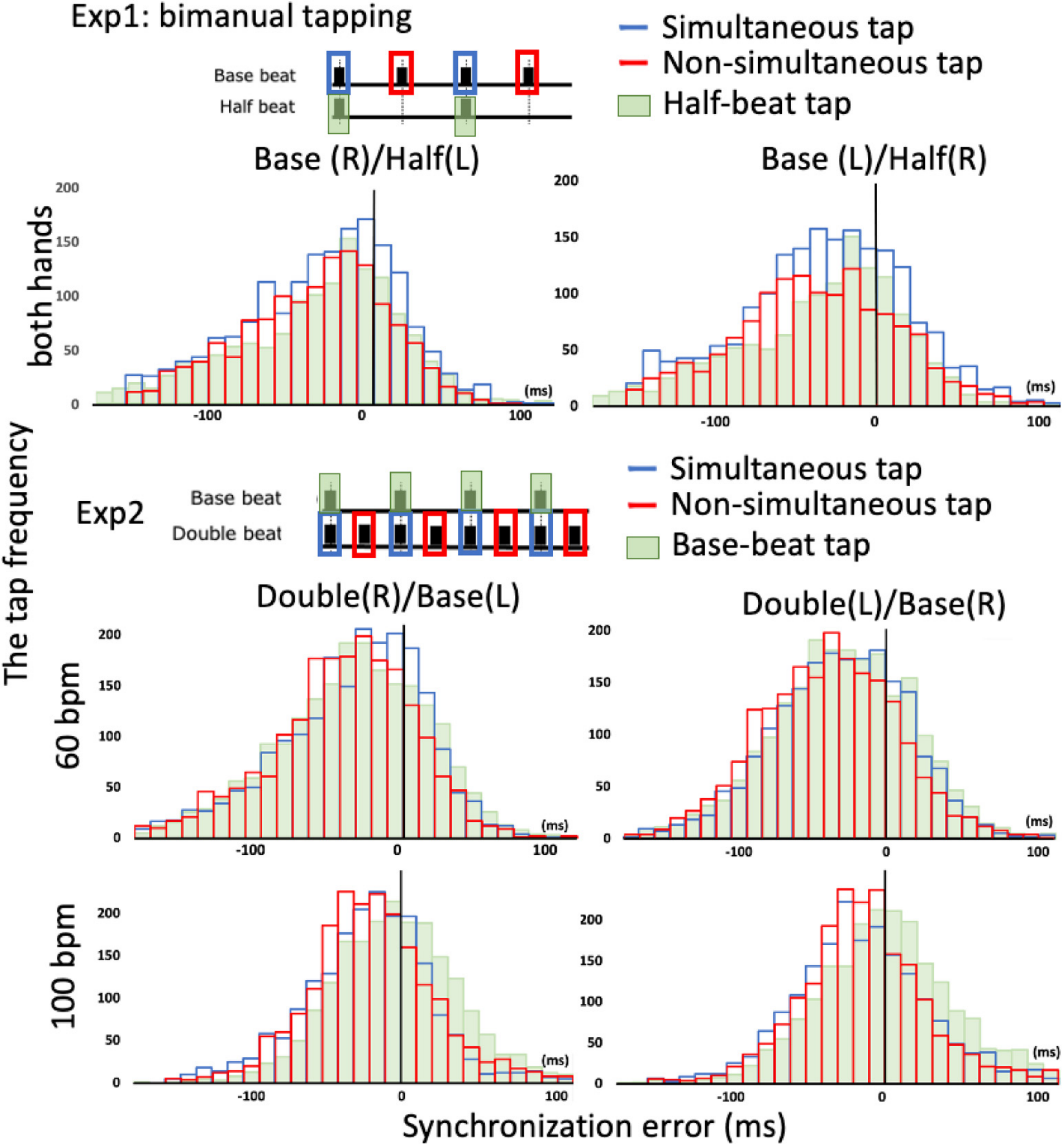
The upper two panels show the distribution of synchronization errors (SEs) under the bimanual tapping condition in Experiment 1. The base beat taps were grouped as two taps, simultaneous, and non-simultaneous taps according to whether there was an accompanying concurrent tap. The histogram shows the SE frequencies for the simultaneous (blue), non-simultaneous (red) and half-beat (green) taps. The lower four panels show the distribution of SEs in Experiment 2. The double beat taps were grouped as simultaneous and non-simultaneous taps. The histogram shows the SE frequencies for the simultaneous (blue), non-simultaneous (red) and base beat (green) taps.

The distribution of the mean SEs for each hand–beat pattern in all participants in Experiment 1 is shown in Figure 3. The upper panels show the SE distribution at the moment of tap-down using two hands (−32.53 ± 30.22 ms; blue bars), and at the moment of tap-down using one hand (29.30 ± 32.15 ms; red bars), under the base-beat condition. The mean SE under the half beat condition (−15.04 ± 20.57 ms; green bars) was half that of the two base beat taps. The SEs under these conditions were distributed ahead of the onset of the auditory pacing signal, demonstrating negative mean asynchrony.

One-way ANOVA was performed to compare the mean SEs of simultaneous, non-simultaneous and half-beat taps, with hand (left vs. right), beat pattern (base vs. half) and tapping (both vs. single) collapsed across groups. The ANOVA revealed a significant main effect of tapping (*F* (2,30) = 19.56, *p* < 0.001, η_p_^2^ = 0.566). Multiple comparisons using the Bonferroni method revealed that the SE of half-beat tapping was smaller than the SEs of the simultaneous and non-simultaneous two taps; *t* (15) = 5.43, *p* < 0.001, r = 0.814; single hand tap (base beat) vs. half beat: *t* (15) = 4.16, *p* = 0.002, r = 0.732). No difference was found between simultaneous and non-simultaneous tapping SEs; *t* (15) = 1.53, *p* = 0.147, r = 0.367).

### Discussion

The purpose of Experiment 1 was to examine whether handedness or beat pattern determined the difference in negative mean asynchrony between the right and left hands. The most important finding was that the SE for the base beat was larger in the negative direction than that observed with the half beat. This finding supports the beat-pattern hypothesis, suggesting that the beat pattern contributed to the greater negative mean asynchrony in the right hand tapping over the other effectors in Fujii et al. (2011). If the handedness hypothesis were correct, the negative mean asynchrony of the right hand should have been consistently smaller regardless of the hand–beat assignments. Thus, the pattern of the results did not support the handedness hypothesis. The comparison of the SEs produced by bimanual and unimanual tapping in Experiment 1 revealed no difference between the conditions, indicating that tapping with two hands does not necessarily increase SE. In other words, the factor that increases the SE is not the number of effectors, but rather the beat pattern itself.

One-way ANOVA revealed two important findings. First, the SE of the half-beat tap was smaller than those of the simultaneous and half-beat taps. Moreover, the simultaneous and half-beat taps were not synchronous, such that the half-beat tap preceded the simultaneous tap. Second, and more importantly, no difference was found between the SEs of the non-simultaneous and simultaneous taps under the base-beat condition, indicating that participants tapped two beat patterns at regular intervals with two hands or one hand under the base-beat condition.

The results of Experiment 1 are consistent overall with the beat-pattern hypothesis. Nonetheless, it remains unclear whether the beat pattern with a shorter inter-onset interval (base beat) produced the beat pattern with a longer inter-onset interval (half beat) or whether the beat pattern with an inter-onset interval identical to the metronome reference signal was tapped earlier than the other beat (i.e., half beat). We designed Experiment 2 to help distinguish between these two alternative explanations.

## Experiment 2

The aim of Experiment 2 was to determine whether the beat pattern with a shorter inter-onset interval (base beat) was the result of the beat pattern with a longer inter-onset interval (half beat), or whether the beat pattern with an inter-onset interval identical to the metronome reference signal was tapped earlier than the other beat (i.e., half beat). To achieve this, we replaced the half beat used in Experiment 1 with a double beat in which two taps were required during the base beat interval. We reasoned that if tapping a beat identical to the auditory pacing signal produced a larger SE, then tapping the base beat should produce a larger SE than tapping the double beat. Alternatively, if tapping at a shorter interval was the critical factor, then the SE should be larger for a double beat than for the base beat. Furthermore, we included a 100 bpm tempo condition to extend our findings.

### Method

Sixteen Hokkaido University undergraduate and graduate students (mean age = 20.63 years, SD = 2.31, male:female = 9:5) participated in the present study. All of the participants had not taken musical training for more than 3 years, and they were right-handed.

Experiment 2 did not include a single-hand tapping task, and the half beat was replaced with a double beat (Figure 1C). Participants tapped the base beat with one hand and tapped twice per the base beat with the other hand as the double beat. In addition, the participants completed two blocks of two tempos (60 and 100 bpm, in that order), as in Fujii et al. (2011); the hand–beat assignment was reversed in the second block.

### Results

**Figure 4.**
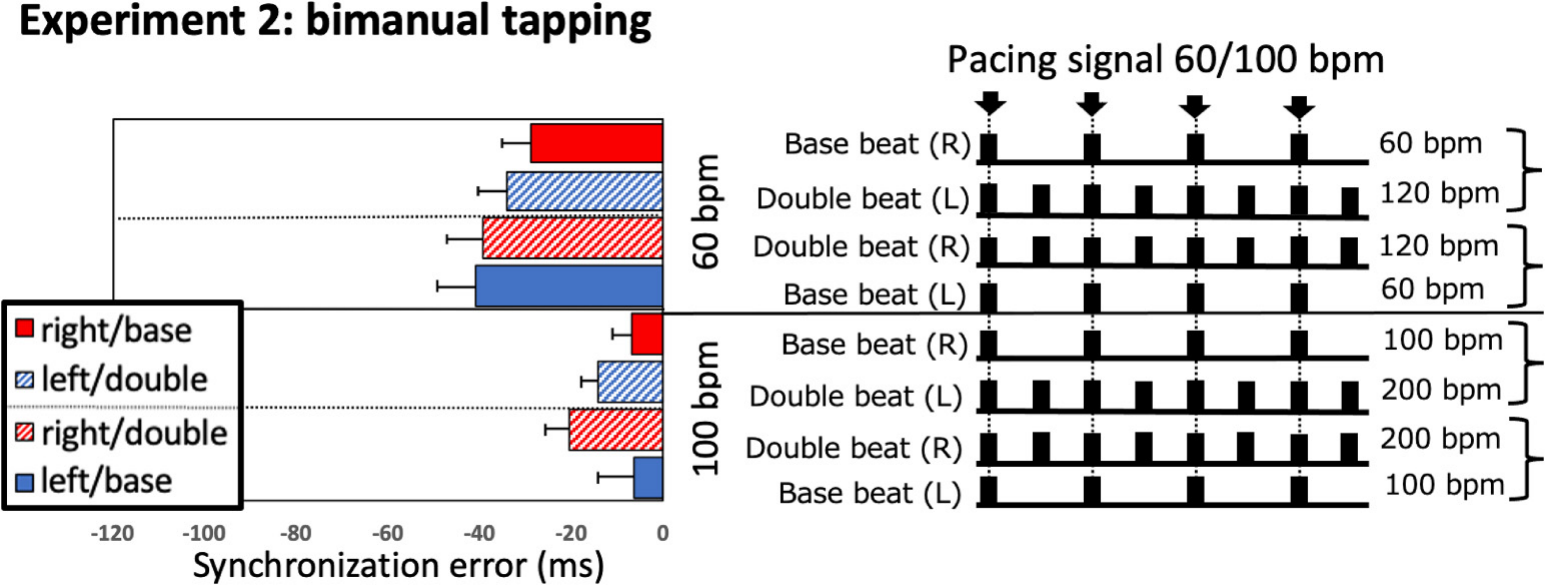
Synchronization errors (SEs, ms) in Experiment 2 left: SEs as a function of hand (both or single) and beat (base or half) for each effector (left or right). The error bars represent the standard error of the average SE. Right: Beat patterns corresponding to the bars in the left panel. Arrows indicate the onset of the pacing signal. The thick bars indicate the moment the participants tapped the base beat at a rate of 60 or 120 bpm (and the double beat at a rate of 100 or 200 bpm) in response to the 60 or 100 bpm pacing signal.

All participants completed four bimanual tapping tasks under two tempo conditions. As shown in Figure 4, participants performed two beat combinations at the 60 bpm tempo: the base beat using the right hand (SE: −28.91 ± 25.17 ms [mean ± standard deviation]) and double beat using the left hand (SE: −34.32 ± 24.34 ms), or the base beat using the left hand (SE: −41.15 ± 33.24 ms) and double beat using the right hand (SE: −39.52 ± 30.20 ms). Under the 100 bpm tempo condition, participants performed the base beat using the right hand (SE: −7.12 ± 15.88 ms) and double beat using the left hand (SE: −14.32 ± 13.91 ms), or the base beat using the left hand (SE: −6.34 ± 30.77 ms) and double beat using the right hand (SE: −20.62 ± 20.17 ms).

A three-way repeated-measures ANOVA with hand (right vs. left), beat pattern (double beat vs. base beat), and tempo (60 vs. 100 bpm) as within-participant factors and SE as the dependent variable revealed significant main effects of tempo (*F* (1,15) = 20.59, *p* < 0.001, η_p_^2^ = 0.579) and beat pattern (*F* (1,15) = 6.09, *p* = 0.026, η_p_^2^ = 0.289), but no main effect of hand (*F* (1,15) < 0.01, *p* = 0.997, η_p_^2^ < 0.001).

The distribution of the SEs for each hand–beat pattern for all participants in Experiment 2 are shown in Figure 3. The lower panels show the distribution of SEs at the moment two hands tapped down (−32.53 ± 30.22 ms; blue bars) and one hand tapped down (−29.30 ± 32.15 ms; red bars) under the base beat condition. The half beat SE (−15.04 ± 20.57 ms; green bars) was half the mean SE of the two other base beat taps. The SEs under these conditions were distributed ahead of the onset of the auditory pacing signal, demonstrating negative mean asynchrony.

The distribution of SEs for each hand–beat pattern in all participants in Experiment 2 is shown in Figure 3. The lower panels show the distribution of SEs at the moment two hands tapped down (−24.59 ± 16.89 ms; blue bars) and the moment one hand tapped down (−27.45 ± 18.56 ms; red bars) under the double beat condition. The base beat (19.24 ± 16.31 ms; green bars) was the mean SE of the two double-beat taps. The SEs under these conditions were distributed ahead of the onset of the auditory pacing signal, demonstrating negative mean asynchrony.

### Discussion

The purpose of Experiment 2 was to examine whether the beat pattern with a shorter inter-onset interval (base beat) was tapped earlier than the beat pattern with a shorter inter-onset interval (double beat) or whether the beat pattern with the same inter-onset interval as the auditory pacing signal was tapped earlier than the other beat. The comparison of SEs between the critical conditions showed that the SE of the double-beat tapping was greater than that of the base beat tapping. Importantly, the findings of Experiments 1 and 2 were consistent in that the SEs were determined by the beat patterns. However, the beat pattern producing greater negative SEs was not the beat pattern that was identical to the auditory pacing signal but rather the beat pattern tapped with a relatively shorter interval.

The finding that larger negative SEs occurred when participants tapped a beat with a relatively shorter interval using two hands indicates that the shorter-interval beat pattern produced larger negative SEs than the longer interval beat. This finding cannot be explained by Weber's law because the shorter interval beat pattern produced a larger negative SE at 100 bpm. However, these findings do not necessarily indicate that the shorter-interval beat pattern produced larger SEs than the longer interval because the SEs under the 100 bpm condition were smaller than those under the 60 bpm, in accordance with Weber's law.

## General Discussion

The present study examined whether the exaggerated negative mean asynchrony in tapping that occurred in one hand relative to the other hand reported in Fujii et al. (2011) was due to the handedness of participants or to the beat pattern used in their study. Fujii et al.’s (2011) finding was unique in that the SEs across hands had never been observed in a task involving bimanual tapping with an identical beat pattern for the hands. Other studies, however, have reported comparable asynchrony errors between hands in general (e.g., Aschersleben and Prinz, 1995). The reasons for the difference in negative mean asynchrony between the two hands were not identified in Fujii et al. (2011) because they maintained the same hand–beat assignments throughout the session. The present study resolved this issue by introducing novel manipulations involving the hand–beat assignments. The comparison between the half beat and the base beat shows that the base beat produced a larger negative SE than the half beat. The comparison between unimanual and bimanual tapping revealed that bimanual tapping did not increase the SE, and that the SEs were determined by beat intervals. Our finding that beat patterns with relatively shorter intervals resulted in larger negative SEs was bolstered by the findings of Experiment 2, which used a double beat pattern in which participants tapped twice per auditory pacing signal. The findings of Experiment 2 showed that a relatively shorter interval beat between hand patterns produced larger negative SEs.

The finding that beat patterns tapped at different intervals produced different SEs, indicates that the hands tapped independently of each other (Yamanishi et al., 1980; Vorberg & Hambuch, 1984), because the asynchrony between hands occurred consistently and systematically across both experiments. It may be that two different systems control the tapping of the right and left hands independently, and thus produce different SEs. However, it should be noted that our findings can also be interpreted in terms of a central timing system. Such a system would control right and left hand tapping by integrating the two beat patterns into a common time base despite the asynchrony (Deutsch, 1978; Klapp, 1979; Lang et al., 1990). In that case, the tapping would be asynchronous with the pacing signal because the participants would erroneously perceive the integrated pattern (resulting from the two asynchronous beats) and pacing signal. Elliott et al. (2006) referred to this erroneous perception as perceptual synchrony. The perceptual synchrony is fed back to the central timing system, producing different SEs between hands^2^. Although evidence for integration has been reported in musicians (Lang et al., 1990), our study may be the first to find such integration in a non-musician population.

Before concluding, the differences between the population examined by Fujii et al. (2011) and the present study should be discussed. Specifically, musicians participated in Fujii et al. (2011), whereas non-musicians participated in the present study. The difference in SEs between the non-musicians in the present study (Experiment 1, 60 bpm condition) and the musicians in Fujii et al. (2011) in the bimanual tapping task was approximately 70 ms. This is seven times greater than that found in a previous study by Aschersleben (1994), which found that the SEs of non-musicians were 10 ms greater than those of musicians in the negative direction on a unimanual tapping task. Other researchers (Aschersleben, 2002; Repp, 1999, 2003) have also reported similar differences in SEs between non-musicians and musicians. The differences between our study of a non-musician cohort and that of Fujii et al., which included musicians, suggest that musical experience improves the temporal accuracy of bimanual tapping; however, further studies comparing musician and non-musician populations are needed to clarify this issue.

In summary, we found that differences in beat patterns determined SEs. Beat patterns with relatively shorter intervals between hand patterns produced larger negative SEs. The SEs did not differ between the bimanual and unimanual tapping conditions suggesting that tapping with two hands does not necessarily increase SE. In other words, the factor that increases SE is not the number of effectors, but the beat pattern itself. Our finding that bimanual tapping of different beat patterns produces different SEs suggests that the concept of a central timing system may need to be revised.

## Author’s Note

The authors thank an anonymous reviewer for suggesting the possibility.
